# Supplementary material for: Estimating 24-Hour Sodium Excretion from Spot Urine Samples in Chinese Adults: Can Spot Urine Substitute 24-Hour Urine Samples?
Source: Nutrients. 2020 Mar 18;12(3):798. doi: 10.3390/nu12030798 (PMC7146571; doi:10.3390/nu12030798)
Supplement: Supplementary file 1 [file nutrients-12-00798-s001.pdf]

Table S1. Equations used in the estimation of 24-h sodium excretion from spot urine.

Method	Formula expression
Kawasaki	Male:$16.3\times[Na_{su}/Cr_{su}\times1/10\times(7.39\times height+15.12\times weight -12.63\times age-79.9)]^{0.5}$
	Female:$16.3\times[Na_{su}/Cr_{su}\times1/10\times(5.09\times height+8.58\times weight -4.72\times age-74.95)]^{0.5}$
Tanaka	$21.98\times[Na_{su}/Cr_{su}\times1/10\times(16.14\times height+14.89\times weight-2.04\times age-2244.45)]^{0.392}$
INTERSALT1	Male:$(0.46\times Na_{su}+25.46)-2.75\times Cr_{su}-0.13\times K_{su}+4.10\times BMI +0.26\times Age$
	Female:$(0.34\times Na_{su}+5.07)-2.16\times Cr_{su}-0.09\times K_{su}+ 2.39\times BMI+ 2.35\times Age-0.03\times Age^2$
INTERSALT2	Male:$(0.45\times Na_{su}+23.51)-3.09\times Cr_{su}+4.16\times BMI+0.22\times Age$
	Female:$(0.33\times Na_{su}+3.74)-2.44\times Cr_{su}+2.42\times BMI+2.34\times Age-0.03\times Age^2$
Toft	Male:$33.56\times[Na_{su}/Cr_{su}\times1/10\times(-7.54\times Age+14.15\times weight+ 3.48\times height+423.15)]^{0.345}$
	Female:$52.65\times[Na_{su}/Cr_{su}\times1/10\times(-6.13\times Age+9.97\times weight+ 2.45\times height+342.73)]^{0.196}$
Whitton	Male:$88.66+0.55\times Na_{su}-1.34\times Cr_{su}-1.05\times K_{su}-0.87\times Age+ 2.10\times BMI +39.30$
	Female:$88.66+0.55\times Na_{su}-1.34\times Cr_{su}-1.05\times K_{su}-0.87\times Age+ 2.10\times BMI$

Note: Na_{su}, Spot urinary sodium; K_{su}, Spot urinary potassium; Cr_{su}, Spot urinary creatinine; The units of concentration of Na_{su}, K_{su} were all mmol/L, Cr_{su} was mg/dL. Weight and height were kg and cm.

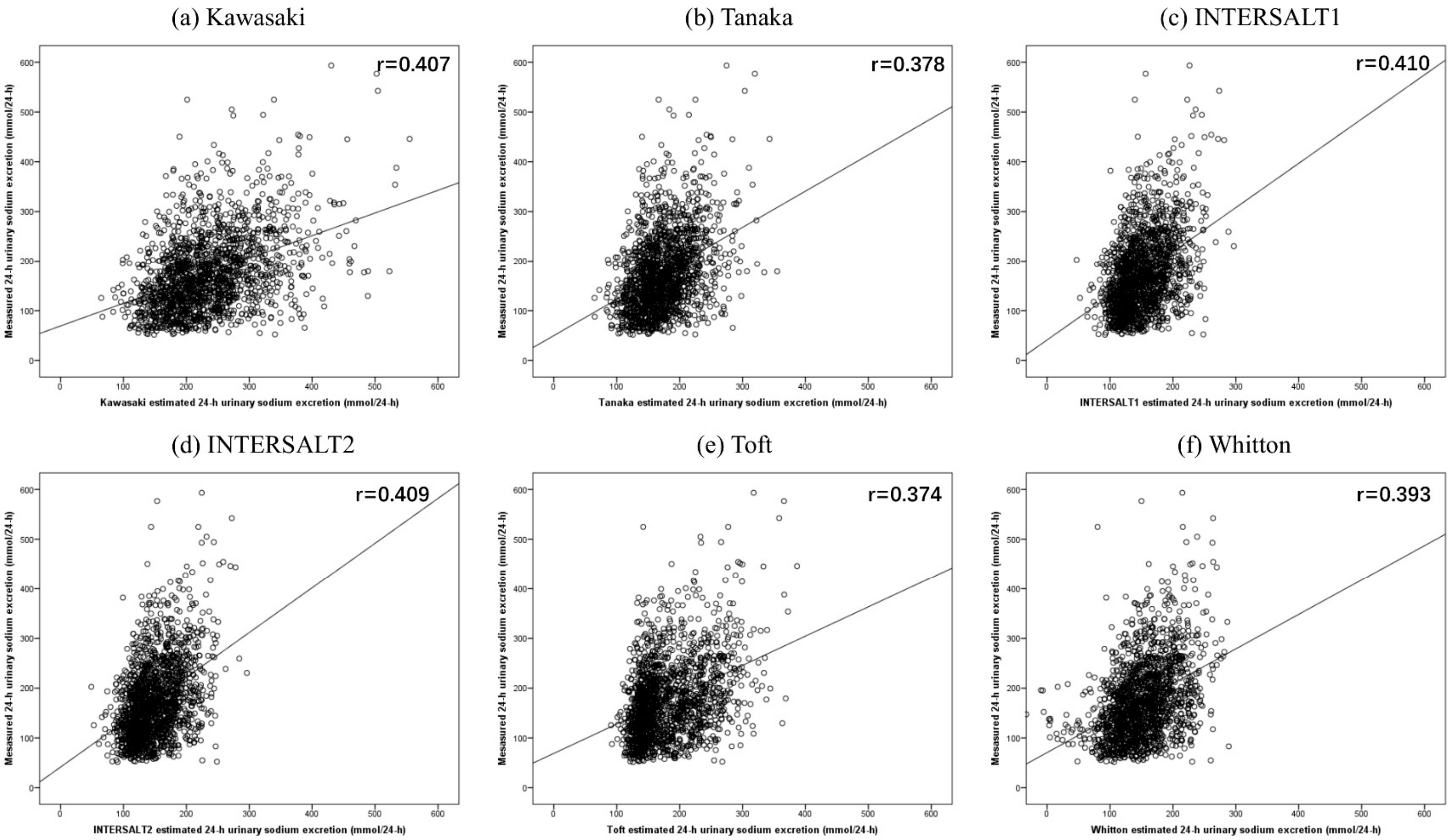

Figure S1. Scatter plots measured 24-hUNa excretion vs. Kawasaki (a), Tanaka (b), INTERSALT1 (c), INTERSALT2 (d), Toft (e) and Whitton (f) methods estimated 24-hUNa excretion. The real line was the liner regression line of the scatters.

Table S2. Misclassification of the six predicted formulas at individual level, n(%).

	Conversion of salt intake by 24-hUNa excretion				Total (n=1671)
	<7 g/day (n=423)	7-9.99 g/day (n=473)	10-12.99 g/day (n=373)	≥13 g/day (n=402)	
Kawasaki	406 (96.0)	387 (81.8)	262 (70.2)	107(26.6)	1162 (69.5)
Tanaka	363 (85.5)	234 (49.5)	218 (58.4)	301 (74.9)	1116 (66.8)
INTERSALT1	284 (67.1)	204 (43.1)	265 (71.0)	359 (89.3)	1112 (66.5)
INTERSALT2	268 (63.4)	217 (45.9)	275 (73.7)	366 (91.0)	1126 (67.4)
Toft	400 (94.6)	205 (43.3)	275 (73.7)	241 (60.0)	1121 (67.1)
Whitton	256 (60.5)	254 (53.7)	268 (71.8)	349 (86.8)	1127 (67.4)
